# Post-ACTH peak cortisol response is associated with genotype in children with nonclassic congenital adrenal hyperplasia

**DOI:** 10.3389/fendo.2026.1745992

**Published:** 2026-03-20

**Authors:** Allie N. Dayno, Marissa J. Kilberg, Erin Gonter, Robert Gallop, Jacob Squicciarini, Carolina Montano, Maria G. Vogiatzi

**Affiliations:** 1 Division of Endocrinology, Children’s Hospital of Philadelphia, Philadelphia, PA, United States; 2 Division of Human Genetics, Children’s Hospital of Philadelphia, Philadelphia, PA, United States

**Keywords:** 21-hydroxylase deficiency, adrenal insufficiency (AI), congenital adrenal hyperplasia (CAH), cortisol, genotype

## Abstract

**Introduction:**

Suboptimal cortisol levels after ACTH stimulation have been recently reported in approximately 30% of patients with nonclassic congenital adrenal hyperplasia (NCCAH). There is little information on the role of genotype on cortisol secretion in NCCAH. The aim of this study is to investigate the association between genotype (i.e compound heterozygote for one mild and one severe *CYP21A2* variant *vs*. homozygous for two mild variants) and peak cortisol response after ACTH stimulation in a pediatric population with NCCAH.

**Methods:**

Retrospective chart review to identify children with NCCAH who had 1) *CYP21A2* genetic information 2) high dose ACTH stimulation test and 3) serum cortisol measurements by LC-MS/MS. The cohort was divided into 2 groups mild/mild (M/M) genotype (mild variants associated with NCCAH include V281L, P30L, P453S) and mild/severe (M/S) genotype (severe variants associated with classic CAH include gene deletion, Q318X, R356W, In2G, I172N) and peak cortisol concentrations were compared. IRB approved.

**Results:**

The cohort included 23 children – 12 M/M and 11 M/S diagnosed at 6.6 years old (SD 3.6, range 0.25-15). Peak cortisol levels were lower in the M/S group compared to M/M (mean +/- SD, 13.2 +/- 3.3 *vs*. 19 +/- 3.6 mcg/dL, p=0.001). The overall prevalence of adrenal insufficiency (AI) using cortisol cutoff of <15 mcg/dL (based on recent literature) was 9/23 (39%) and was higher in M/S *vs*. M/M (8/11, 73% *vs*. 1/12, 8%, p=0.003).

**Conclusion:**

The data suggest higher rates of suboptimal cortisol response in children with NCCAH who have mild/severe genotype compared to mild/mild.

## Introduction

Congenital adrenal hyperplasia (CAH) due to 21-hydroxylase deficiency encompasses a continuum of clinical severity based on the amount of the residual enzyme activity. It is an autosomal recessive disorder caused by biallelic pathogenic variants in the *CYP21A2* gene, located on the short arm of chromosome 6. Nonclassic CAH (NCCAH), also known as late-onset CAH, is a milder form characterized by 20-50% of 21-hydroxylase enzyme activity compared to 0-2% activity seen in classic CAH. NCCAH is more common than classic CAH, with an estimated prevalence in the US population of ~1:200, and higher rates in certain ethnic groups (1). Individuals with NCCAH may be asymptomatic or present in childhood or adolescence with signs of hyperandrogenism, including hirsutism, acne, body odor, pubic hair, rapid growth, and advanced bone age. The diagnosis of NCCAH is usually made with an elevated morning (before 8am) 17-hydroxyprogesterone (17-OHP) concentration between 200-10,000 ng/dL. A standard high dose or 250 mcg (0.25 mg) ACTH stimulation test with measurement of stimulated 17-OHP concentrations can confirm the diagnosis but is not done consistently in clinical practice (1, 2).

Children with NCCAH are considered low risk for cortisol deficiency and are not routinely prescribed treatment with glucocorticoids. The Endocrine Society clinical practice guidelines recommend glucocorticoid treatment in youth who experience rapid progression of pubarche or bone age advancement or female adolescents with virilization (1). They explain that the risks and benefits of such treatment should be discussed with family given the risks of adrenal suppression and patients with NCCAH should be given the option to stop glucocorticoids once adult height is attained or symptoms improve. Additionally, current guidelines do not recommend screening for adrenal insufficiency (AI) or stress dose steroid coverage in patients with NCCAH. However, recent studies have shown rates of suboptimal peak cortisol on ACTH stimulation ranging from 15-60% (3, 4). At the moment, there is very little and conflicting information about cortisol secretion and genotype in NCCAH (3, 5).

Pathogenic variants in *CYP21A2* can be categorized based on *in vitro* data that corresponds to residual enzyme activity and correlate with disease severity classified as salt wasting (SW), simple virilizing (SV), and NCCAH (6–9). SW and SV are the subtypes of classic CAH in which there is much less enzymatic activity (0-2%) leading to a more severe phenotype consisting of glucocorticoid deficiency with or without mineralocorticoid deficiency. There is high genotype-phenotype concordance in certain variants such as the 30kb deletion, Q318X, R356W (severe mutations associated with classic CAH) and V281L (mild mutation associated with NCCAH) (10). Compound heterozygotes with one mild and one severe variant clinically present as NCCAH since the phenotype is correlated with the mildest mutation; however, they may manifest more severe symptomatology than those who are biallelic for the mild variant. The aim of this study is to investigate the association between genotype and peak cortisol response in a pediatric population with NCCAH.

## Methods

**Figure 1:**
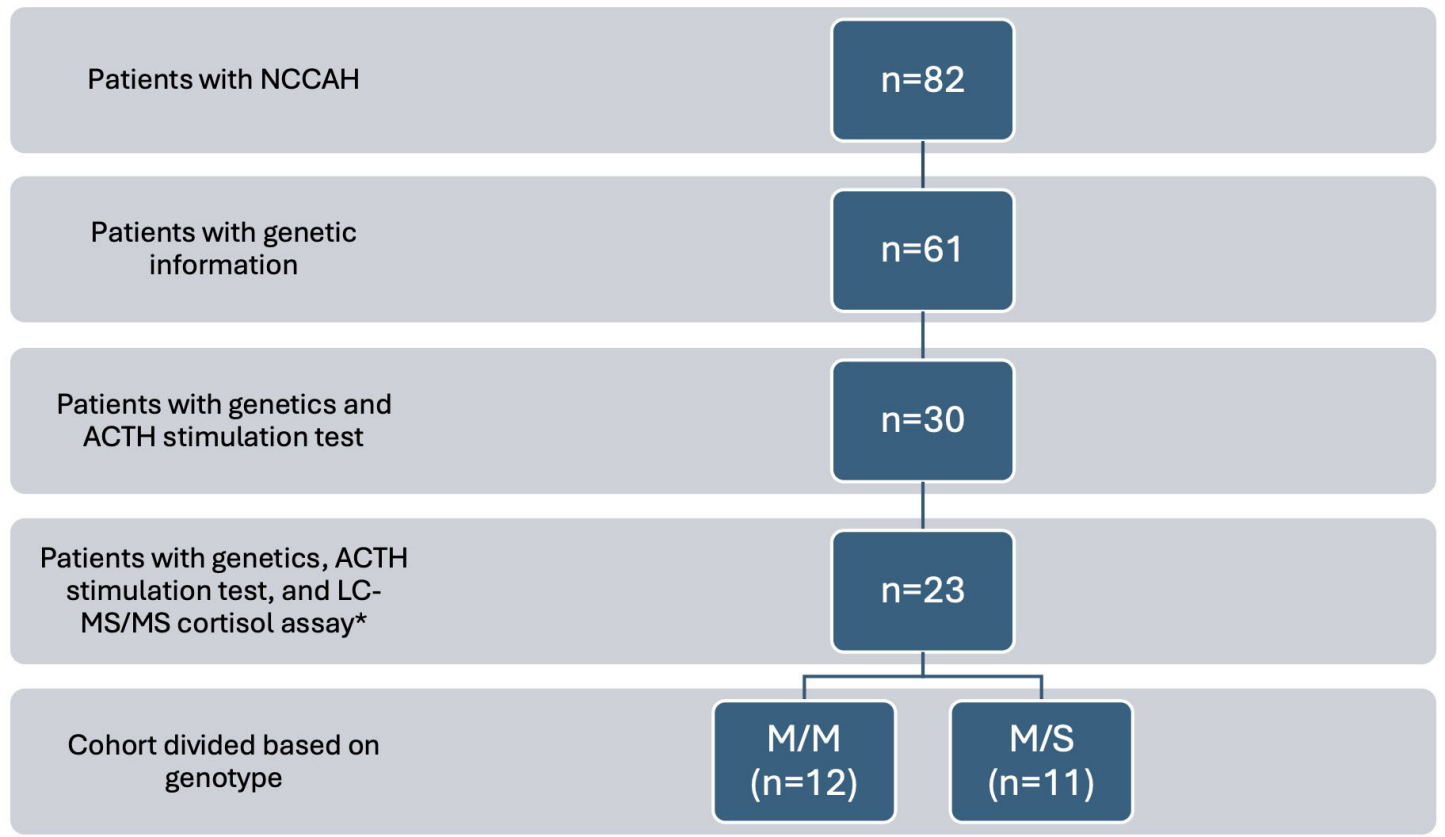
Diagram of cohort selection. Electronic medical records of 82 patients with NCCAH reviewed. Those without genetic information and ACTH stimulation test with cortisol assay by LC-MS/MS were excluded. 23 patients fulfilled with inclusion criteria and were divided based on genotype. *7 patients were excluded from the 30 patients with genetics and ACTH stimulation test (5 had testing done at outside institutions and unable to verify cortisol assay; 2 had cortisol by immunoassay). M/M, mild/mild; M/S, mild/severe.

We conducted an IRB approved retrospective chart review of 82 patients with NCCAH seen at our pediatric endocrinology clinic from 2007-2024 (**Figure 1**). The diagnosis of NCCAH was defined by a morning 17-OHP concentration ≥200 ng/dL, stimulated 17-OHP concentration ≥1,000 ng/dL, and/or confirmatory molecular genetic analysis of *CYP21A2*. In the subset of patients who underwent the high dose ACTH stimulation test, it was performed by obtaining a baseline and 60 minute 17-OHP and cortisol concentration after administration of cosyntropin intravenously (0.015 mg/kg for neonates, 0.125 mg for children younger than 2 years, and 0.25 mg for children older than 2 years). The majority of ACTH stimulation tests were done at our outpatient day medicine unit using the CAH pediatric profile 6 (CAH6) comprehensive screen or CAH pediatric profile 1 (CAH1) panel as the laboratory test. The CAH6 comprehensive screen includes 17-OHP, androstenedione, cortisol, dehydroepiandrosterone (DHEA), deoxycorticosterone (DOC), 11-desoxycortisol, 17-OH pregnenolone, progesterone, and testosterone. The CAH1 panel includes 17-OHP, androstenedione, cortisol, DHEA, and testosterone. Hormone concentrations were measured using LC-MS/MS performed by Esoterix (Labcorp Specialty Testing Group, Calabasas Hills, CA, USA). Clinical presentations leading to evaluation for NCCAH varied widely and included premature adrenarche (most common), family history of CAH, irregular periods, hirsutism, and growth concerns. The study population consisted of patients who met the following criteria: 1) *CYP21A2* genetic information available 2) high dose ACTH stimulation test completed and 3) serum cortisol measurements by LC-MS/MS.

We reviewed electronic medical records of 82 patients (**Figure 1**). 61 patients had *CYP21A2* genetic testing. One patient was excluded despite meeting criteria for NCCAH based on stimulated 17-OHP concentration >1,000 ng/dL since only one V281L variant was identified. 30 patients had both genetic testing and high dose ACTH stimulation test (7 did not have cortisol testing by LC-MS/MS assay). Ultimately, 23 patients fulfilled the inclusion criteria.

The cohort of 23 youth were separated into two groups based on genotype. 12 patients had biallelic mild variants and were classified as mild/mild (M/M). 11 patients were compound heterozygotes for one mild and one severe variant and were classified as mild/severe (M/S). *CYP21A2* mild variants associated with NCCAH included V281L, P453S, P30L. *CYP21A2* severe variants associated with classic CAH included I172N, In2G, 30kb deletion, Q318X, R408H (**Table 1**). One patient had a severe variant and also had 3 variants (c.-126C>T, c.-113G>A, c.-110T>C) in the precoding region of the pseudogene *CYP21A1P* indicative of microgene conversion event where the 3 variants were transferred from the pseudogene to the active gene *CYP21A2*. Promoter variants in *CYP21A2* can lead to reduced transcriptional activities and possibly associated with mild disease; therefore, this patient was categorized as M/S (11). Genetic analysis of *CYP21A2* was performed by commercially available laboratories and included gene sequencing. Specifically, the commercial labs included: Prevention Genetics (long range PCR amplification of the functional *CYP21A2* gene followed by bi-directional sanger sequencing; n=15), Quest (next generation sequencing; n=5), LabCorp (PCR amplification of *CYP21A2* followed by multiplex minigene sequencing with primer extension to detect the 12 most common pathogenic variants – P30L, In2G, 8bp deletion, I172N, I236N, V237E, M239K, V281L, F306+t, Q318X, R356W, P453S; n=2). One infant with NCCAH based on ACTH stimulation testing had prenatal parental carrier testing performed by Myriad Foresight Carrier Screen that revealed both parents carried a copy of V281L variant, and, therefore, the child was presumed to be homozygous for V281L. Suboptimal cortisol peak was defined as using cortisol cutoff of <15 mcg/dL (based on recent literature of LC-MS/MS cortisol assays) (12).

**Table 1:** Genotype characterization of mild (non-classic) variants and severe (salt wasting and simple virilizing) variants based on studies showing *in vitro* activity of *CYP21A2*.

Genotype	HGVS nomenclature	Number of patients	Genotype characterization
V281L/V281L	c.844G>T / c.844G>T (p.Val282Leu)	9	M/M
V281L/P453S	c.844G>T / c.1360C>T (p.Val282Leu / p.Pro454Ser)	3	M/M
V281L/del	c.844G>T / large gene deletion	4	M/S
V281L/R408H	c.844G>T / c.1223G>A (p.Val282Leu / p.Arg409His)	1	M/S
*3 precoding variants/I172N	c.-126C>T, c.-113G>A, c.-110T>C / c.518T>A (p.Ile172Asn)	1	M/S
V281L/Q318X	c.844G>T / c.955C>T (p.Val282Leu / p.Gln319Ter)	2	M/S
V281L/In2G	c.844G>T / c.293-13C>G (p.Val282Leu / I2 splice)	2	M/S
P30L/I172N	c.89C>T / c.518T>A (p.Pro30Leu / p.Ile172Asn)	1	M/S

*3 variants in precoding region of pseudogene (*CYP21A1P*) indicative of microgene conversion. Promoter variants in *CYP21A2* can lead to reduced transcriptional activities and possibly associated with mild disease (11).

M/M, mild/mild; M/S, mild/severe.

STATA 18.0 was used for statistical analysis. The continuous variables are presented as mean and standard deviation. The categorical variables are expressed as numbers and percentages. Demographic data and clinical characteristics between the two groups were analyzed using one independent sample t-test way for continuous variables and Fishers exact test for categorical variables. When significant deviation of the normality of model-based residuals was observed, independent t-test were replaced by Kruskal-Wallis nonparametric test. The number needed to treat (NNT) is calculated as the reciprocal of the difference in prevalence of AI in the M/S group and prevalence of AI in the M/M group (13).

## Results

The cohort included 23 children – 12 (52%) in the M/M group and 11 (48%) in the M/S group (**Table 2**). The mean age of diagnosis in the entire cohort was 6.6 years old (+/-3.6, range 0.25–15 years). Within the entire cohort, 5 patients presented due to a family history of CAH or prenatal parental carrier testing (ages ranged from 0.25–5 years). 2 males had a family history of CAH (1 NCCAH and 1 classic CAH). 3 children (2 female, 1 male) had positive parental carrier testing without a known family history of CAH. Most children (16/23) presented with premature adrenarche (one child also had growth concerns in addition to premature adrenarche). Two teenagers presented with irregular periods/hirsutism (one had M/M genotype and one had M/S genotype).

**Table 2:** Demographics and clinical characteristics of Mild/Mild (M/M) and Mild/Severe (M/S) cohorts stratified by gender.

Characteristic	M/M – male (n=4)	M/M – female (n=8)	M/M (n=12)	M/S – male (n=6)	M/S – female (n=5)	M/S (n=11)
Age at diagnosis, mean (SD)	7.7 (1.9)	7.6 (4.1)	7.7 (3.2)	3.8 (3.1)	7.2 (3.8)	5.3 (3.6)
Presentation, n (%)						
a. Premature adrenarche	3 (75)	6 (75)	9 (75)	4 (67)	3 (60)	7 (64)
b. Family history	1 (25)	1 (12.5)	2 (17)	2 (33)	1 (20)	3 (27)
c. Irregular periods	NA	1 (12.5)	1 (8)	NA	1 (20)	1 (9)
Height Z score*, mean (SD)	-0.16 (0.73)	0.06 (1.07)	-0.02 (0.94)	1.56 (1.69)	0.82 (1.15)	1.22 (1.45)
BMI Z score*, mean (SD)	0.72 (0.96)	0.57 (0.90)	0.62 (0.88)	0.86 (1.22)	1.26 (0.78)	1.05 (1.02)
Baseline 17-OHP, mean (SD), ng/dL	699 (755)	909 (583)	839 (619)	919 (638)	2076 (1685)	1445 (1305)
Baseline androstenedione, mean (SD), ng/dL	60 (49)	126 (99)	104 (89)	66 (55)	160 (146)	109 (112)

*Growth parameters at initial evaluation. Bone age information was not included because 5 patients did not have bone age data available to review.

17-OHP, 17-hydroxyprogesterone; NA, not applicable.

**Figure 2:**
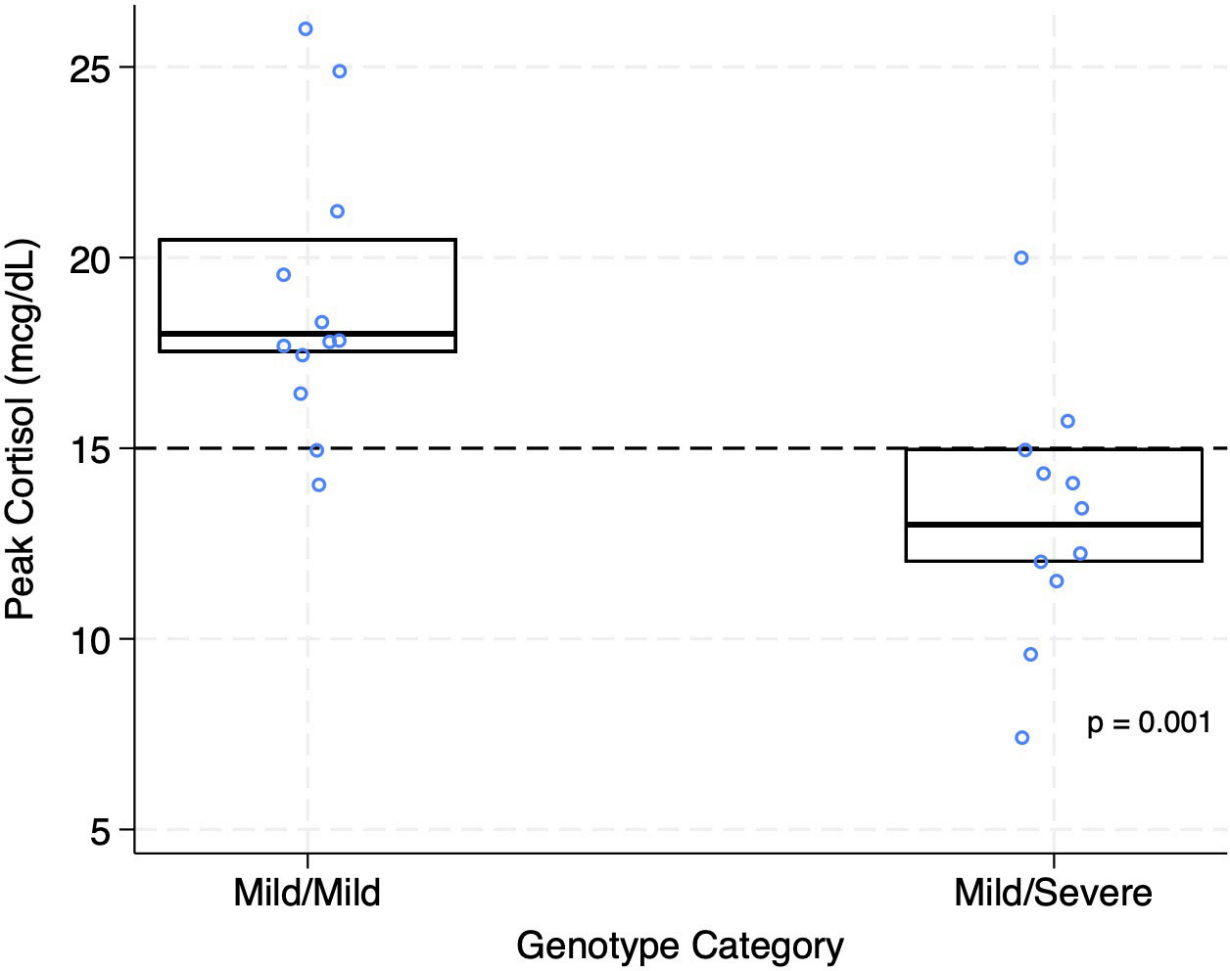
Post-ACTH peak cortisol and genotype. Scatter plots of peak cortisol concentrations after ACTH stimulation grouped according to genotype. Boxes represent the interquartile range, and the horizontal line within each box represents the median. Dots represent individual values. The dashed line indicates the clinical cutoff of 15 mcg/dL for an adequate cortisol response.

**Figure 3:**
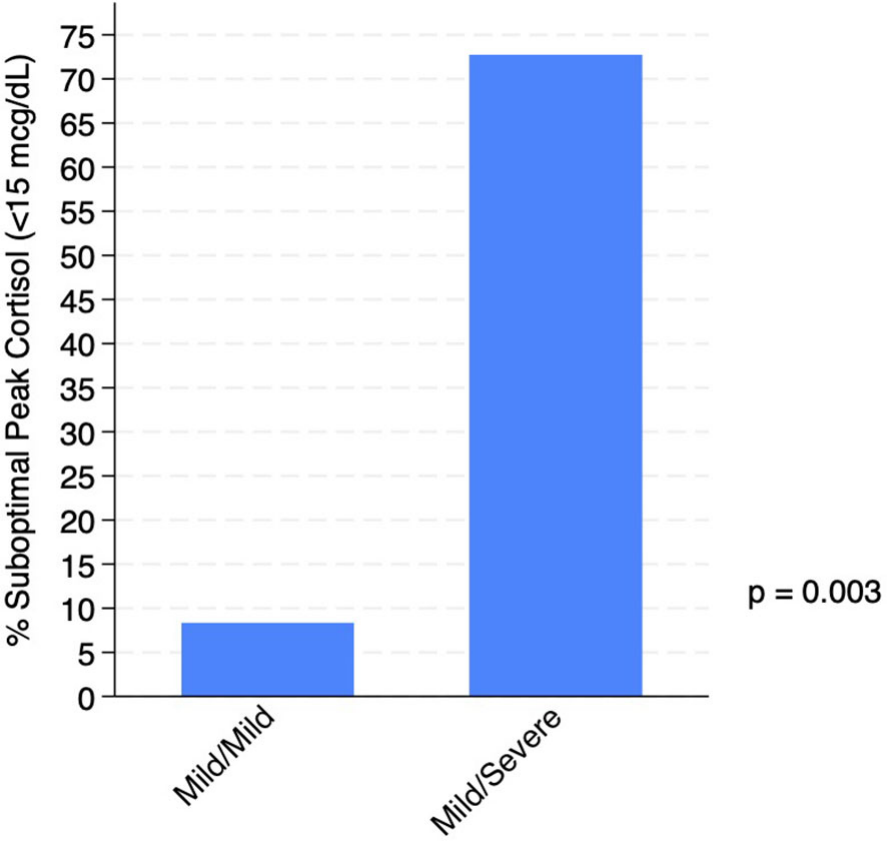
Suboptimal cortisol response and genotype group. Percent of subjects in each genotype group with suboptimal peak cortisol (defined as <15 mcg/dL based on recent literature of LC- MS/MS cortisol assays).

Peak cortisol levels were lower in the M/S group compared to M/M (mean +/- SD, 13.2 mcg/dL +/- 3.3 *vs*. 19 mcg/dL +/- 3.6, p=0.001) (**Figure 2**; **Table 3**). As a measure of clinical significance Cohen’s standardized effect size yielded d=1.69 (95% CI 0.68-2.57) corresponding to a very large effect size. The overall prevalence of AI using a cortisol cutoff of <15 mcg/dL (based on recent literature of LC-MS/MS cortisol assays) was 9/23 (39%) and was higher in M/S *vs*. M/M (8/11, 73% *vs*. 1/12, 8%, p=0.003) (**Figure 3**; **Table 3**). Statistical significance remains when using the traditional cortisol cutoff of <18 mcg/dL. The overall prevalence of AI using a cortisol cutoff of <18 mcg/dL was 13/23 (57%) and was higher in M/S *vs*. M/M (10/11, 91% *vs*. 3/12, 25%, p=0.003). Clinical significance index of number needed to treat (NNT) yielded NNT = 1.55 (95% CI 1.04-2.77) and NNT = 1.52 (95% CI 1.04-2.77) for <15 mcg/dL and <18 mcg/dL cutoffs, respectively, corresponding to very large effect sizes (13).

**Table 3:** Peak cortisol, prevalence of adrenal insufficiency (AI) based on suboptimal cortisol peak <15 mcg/dL, and 17-hydroxyprogesterone concentrations in each genotype group and entire cohort.

Parameter	Mild/Mild (n=12)	Mild/Severe (n=11)	*P* value	Entire cohort (n=23)
Peak cortisol, mean (SD), mcg/dL	19 (3.6)	13.2 (3.3)	0.001	16.2 (4.4)
Prevalence of AI, n (%)	1 (8%)	8 (73%)	0.003	9 (39%)
Stimulated 17-hydroxyprogesterone, mean (SD), ng/dL	3764 (1515)	6176 (2269)	0.008	4918 (2238)

## Discussion

The data suggest higher rates of suboptimal peak cortisol response after ACTH stimulation testing in children with NCCAH who have M/S genotype compared to M/M. Despite the small sample size, very large effects were seen in our investigations. The majority of children with M/S genotype had a suboptimal cortisol response to ACTH stimulation (i.e. <15 mcg/dL), indicating that children who are compound heterozygous with one mild and one severe variant may behave clinically different than those with biallelic mild variants. Traditionally, NCCAH has been seen as a uniform condition consisting of a mild phenotype without the need for lifelong glucocorticoid treatment. These results reveal the importance of viewing NCCAH as a spectrum of clinical severity – analogous to CAH as a whole (6).

Our results are consistent with recent literature that reports suboptimal cortisol response to ACTH stimulation in some patients with NCCAH (3–5). The specific rates vary from 15-60% and likely reflect differences in methodology, cortisol assays and cutoffs. Specifically, a study by Stoupa et al. observed that 60% of their participants had an inadequate cortisol response using the cutoff of 18 mcg/dL but did not explore associations with genotype (4). The relationship between genotype and cortisol response was addressed in only two studies, which yield conflicting results (3, 5). While Dorr et al. failed to observe a correlation, Koren et al. demonstrated that M/S genotype was associated with lower peak cortisol levels compared to M/M group in a group of 106 patients with NCCAH (3, 5). Our study, focused on a pediatric cohort with NCCAH, also identified a strong correlation of the M/S genotype and suboptimal cortisol response.

The clinical implications of an inadequate cortisol response in children and adolescents with NCCAH remain unclear, and ACTH stimulation testing is subject to several limitations. Clinical manifestations of adrenal insufficiency are uncommon (1). A chart review of the nine children with suboptimal peak cortisol concentrations in this cohort revealed no documented clinical manifestations of adrenal insufficiency at baseline or during procedures (e.g. colonoscopy and tonsillectomy) based on clinic and procedure notes in the electronic health record.

Furthermore, established cortisol cutoffs after ACTH stimulation are somewhat arbitrary and revised with the introduction of newer monoclonal immunoassays and LC-MS/MS (14). The peak cortisol concentration in our cohort ranged from 7.5 to 26 mcg/dL. There are potential confounding factors in regards to ACTH stimulation testing, including cortisol cutoffs, timing of collection (30 minute *vs*. 60 minute collection), sampling errors, and current medications. In this study, we used LC-MS/MS for serum cortisol measurements and the cutoff of 15mcg/dL to define appropriate cortisol response, as suggested by recent literature. Previous studies investigating cortisol reserve in NCCAH used polyclonal antibody assays and the traditional cutoff of 18 mcg/dL (500 nmol/L) (3–5). Nonetheless, we observed a strong correlation of genotype and cortisol response for both cutoffs of 15 and 18mcg/dL. Using a cortisol cutoff of 18 mcg/dL rather than 15 mcg/dL increases the prevalence of suboptimal peak cortisol from 8 to 25% in the M/M group *vs*. 73 to 91% in the M/S group. There are still significantly more people with suboptimal cortisol levels in the M/S group compared to M/M even when using the higher cutoff value. Given the potential of symptomatic adrenal insufficiency under a major physiologic stress, it is our practice to provide stress coverage for NCCAH children with suboptimal cortisol response. However, we acknowledge that further studies are needed to address this.

CAH due to 21-hydroxylase deficiency represents a clinical continuum, and studies have shown strong genotype-phenotype correlation with specific pathogenic variants. Nevertheless, specific variants or variant combinations have been associated with phenotypic variability, particularly within the classical CAH spectrum (7, 15). Our study demonstrates that there is also phenotypic variability in patients with NCCAH, influenced in part by the M/S genotype. Specifically, children with NCCAH who are compound heterozygous for one mild and one severe variant may benefit from ACTH testing to assess cortisol response and determine the need for possible treatment with stress dose glucocorticoids.

Limitations of this investigation include a small sample size and retrospective design. Despite the small size, for the large effects detected, Kraemer & Kupfer illustrate we have over 80% power for such effects (13). More studies are needed to confirm these findings and determine the association between genotype and other disease outcomes, such as growth abnormalities, in these children.

Despite its high prevalence, evaluation of infants and children with NCCAH is not fully standardized. Screening for adrenal insufficiency and genetic testing are not routinely recommended by the latest Endocrine Society guidelines (1). Furthermore access to ACTH stimulation testing is variable. Patients with suspected NCCAH benefit from ACTH testing to confirm diagnosis by peak 17-OHP concentration and assess cortisol response to determine need for stress dose coverage. Our findings indicate that genotype influences cortisol response in NCCAH and support the use of genetic testing for risk stratification in children with NCCAH. This data, collectively with recent literature, support the need for genotyping to guide clinical management in NCCAH.

## Data Availability

The original contributions presented in the study are included in the article/supplementary material. Further inquiries can be directed to the corresponding author.

## References

[1] Speiser PW Arlt W Auchus RJ Baskin LS Conway GS Merke DP . Congenital adrenal hyperplasia due to steroid 21-hydroxylase deficiency: An Endocrine Society* clinical practice guideline. J Clin Endocrinol Metab. (2018) 103:4043–88. doi: 10.1210/jc.2018-01865, PMID: 30272171 PMC6456929

[2] Nimkarn S Gangishetti PK Yau M . “ 21-Hydroxylase-Deficient Congenital Adrenal Hyperplasia.” In: Adam MP Bick S Mirzaa GM , editors. GeneReviews® [Internet]. Seattle (WA): University of Washington, Seattle 1993–2026. (2002). 20301350

[3] Dorr HG Schulze N Bettendorf M Binder G Bonfig W Denzer C . Genotype-phenotype correlations in children and adolescents with nonclassical congenital adrenal hyperplasia due to 21-hydroxylase deficiency. Mol Cell Pediatr. (2020) 7:8. doi: 10.1186/s40348-020-00100-w, PMID: 32647925 PMC7347723

[4] Stoupa A Gonzalez-Briceno L Pinto G Samara-Boustani D Thalassinos C Flechtner I . Inadequate cortisol response to the tetracosactide (Synacthen®) test in non-classic congenital adrenal hyperplasia: An exception to the rule? Horm Res Paediatr. (2015) 83:262–7. doi: 10.1159/000369901, PMID: 25677445

[5] Koren I Weintrob N Kebesch R Majdoub H Stein N Naor S . Genotype-specific cortisol reserve in a cohort of subjects with non-classic congenital adrenal hyperplasia (NCCAH). J Clin Endocrinol Metab. (2023) 109(3):852–7. doi: 10.1210/clinem/dgad546, PMID: 37715965

[6] Krone N Arlt W . Genetics of congenital adrenal hyperplasia. Best Pract Res Clin Endocrinol Metab. (2009) 23:181–92. doi: 10.1016/j.beem.2008.10.014, PMID: 19500762 PMC5576025

[7] Speiser PW Dupont J Zhu D . Disease expression and molecular genotype in congenital adrenal hyperplasia due to 21-hydroxylase deficiency. J Clin Invest. (1992) 90:584–95. doi: 10.1172/JCI115897, PMID: 1644925 PMC443137

[8] Krone N Rose IT Willis DS Hodson J Wild SH Doherty EJ . Genotype-phenotype correlation in 153 adult patients with congenital adrenal hyperplasia due to 21-hydroxylase deficiency: Analysis of the United Kingdom Congenital adrenal Hyperplasia Adult Study Executive (CaHASE) cohort. J Clin Endocrinol Metab. (2013) 98:E346–54. doi: 10.1210/jc.2012-3343, PMID: 23337727 PMC3651585

[9] Claahsen-van der Grinten HL Speiser PW Ahmed SF Arlt W Auchus RJ Falhammar H . Congenital adrenal hyperplasia-current insights in pathophysiology, diagnostics, and management. Endocr Rev. (2022) 43:91–159. doi: 10.1210/endrev/bnab016, PMID: 33961029 PMC8755999

[10] New MI Abraham M Gonzalez B Dumic M Razzaghy-Azar M Chitayat D . Genotype-phenotype correlation in 1,507 families with congenital adrenal hyperplasia owing to 21-hydroxylase deficiency. Proc Natl Acad Sci USA. (2013) 110:2611–6. doi: 10.1073/pnas.1300057110, PMID: 23359698 PMC3574953

[11] Araujo RS Mendonca BB Barbosa AS Lin CJ Marcondes JA Billerbeck AE . Microconversion between CYP21A2 and CYP21A1P promoter regions causes the nonclassical form of 21-hydroxylase deficiency. J Clin Endocrinol Metab. (2007) 92:4028–34. doi: 10.1210/jc.2006-2163, PMID: 17666484

[12] Javorsky BR Raff H Carroll TB Algeciras-Schimnich A Singh RJ Colón-Franco JM . New cutoffs for the biochemical diagnosis of adrenal insufficiency after ACTH stimulation using specific cortisol assays. J Endocr Soc. (2021) 5(4):bvab022. doi: 10.1210/jendso/bvab022, PMID: 33768189 PMC7975762

[13] Kraemer HC Kupfer DJ . Size of treatment effects and their importance to clinical research and practice. Biol Psychiatry. (2006) 59:990–6. doi: 10.1016/j.biopsych.2005.09.014, PMID: 16368078

[14] Charoensri S Auchus RJ . A contemporary approach to the diagnosis and management of adrenal insufficiency. Endocrinol Metab (Seoul). (2024) 39:73–82. doi: 10.3803/EnM.2024.1894, PMID: 38253474 PMC10901672

[15] Concolino P Falhammar H . Genetics in congenital adrenal hyperplasia due to 21-hydroxylase deficiency and clinical implications. J Endocr Soc. (2025) 9:bvaf018. doi: 10.1210/jendso/bvaf018, PMID: 39911519 PMC11795198

